# Assessment of tannery effluents quality treated by electrocoagulation and ozonation: Physicochemical and ecotoxicological characterization

**DOI:** 10.1371/journal.pone.0328654

**Published:** 2025-07-22

**Authors:** Edwar Aguilar-Ascón, Liliana Marrufo-Saldaña, Julio Barra-Hinojosa, Robert Buleje-del-Carpio

**Affiliations:** 1 Instituto de Investigación Científica, Grupo de Investigación en Tecnologías, Exponenciales, Estudios Generales, Universidad de Lima, Lima, Perú; 2 Centro de Innovación Productiva y Transferencia Tecnológica del Cuero, Calzado e Industrias Conexas (CITEccal Lima), Instituto Tecnológico de la Producción (ITP), Lima, Perú; National Research and Innovation Agency, INDONESIA

## Abstract

Tannery effluents are characterized by their high toxicity and complex pollutant load, posing significant risks to aquatic ecosystems. Although conventional treatment processes often achieve regulatory standards for pollutant concentrations, they do not necessarily guarantee the reduction of effluent toxicity. This study aimed to evaluate the quality of tannery effluents treated by electrocoagulation (EC) and the combined electrocoagulation-ozonation (ECO) process, while analyzing the associated toxicity reduction, in order to determine the suitability of these technologies for application and ensure environmental protection of receiving water bodies. For this purpose, tannery wastewater was treated sequentially using an electrocoagulation reactor followed by an ozonation system, yielding three sample types: raw (C), electrocoagulation-treated (EC), and electrocoagulation-ozonation treated (ECO). Physicochemical parameters were measured, toxicity was assessed through bioassays with Lactuca sativa and Eisenia fetida, and chemical changes were analyzed using FTIR spectroscopy.EC achieved 96.4% removal of total suspended solids (TSS), 30.9% of chemical oxygen demand (COD), and 99% of chromium, while ozonation further removed 10% of COD and 99% of sulfides. Toxicity assays indicated a reduction from 23.89 toxicity units (TUs) in the raw effluent to 8.32 TUs after EC and 11.12 TUs after ECO. The slight increase in toxicity after ozonation was associated with elevated ammoniacal nitrogen levels and the formation of new functional groups, as evidenced by the FTIR spectrum. Despite significant pollutant removal, the results highlight that treated effluents may still present residual toxicity, emphasizing the need for complementary treatment strategies to achieve true environmental safety.

## Introduction

The tannery industry transforms hides into leather, a material widely used in the manufacture of footwear, clothing, and other products. Among the byproducts generated by this industry are solid waste and liquid effluents. The latter are recognized for their high pollutant load, including heavy metals such as chromium, ammonium, sulfides, persistent organic compounds, and others, making it necessary to provide adequate treatment before discharge [1,2].

Electrocoagulation (EC) is an electrochemical process based on the in situ generation of coagulant species through the dissolution of metallic electrodes, typically aluminum or iron, under the application of electric current [3,4]. This dissolution releases metal ions that react in the aqueous medium, neutralizing the charges of pollutants and promoting their removal through physicochemical mechanisms [3,5]. The flocs formed during this process are generally large, stable, resistant to acidic media, have low water content, and can be easily separated by filtration systems [6].

During electrocoagulation, four fundamental mechanisms occur simultaneously, accounting for the process’s efficiency. First, electrochemical reactions take place on the surface of the electrodes: at the anode, the metal undergoes oxidation, releasing metal ions such as Al³⁺ or Fe² ⁺ , while at the cathode, water is reduced, generating hydroxide ions (OH⁻) and hydrogen gas (H₂) [7]. Second, the metal ions react with the hydroxide ions to form coagulant species, mainly amorphous metal hydroxides such as Al(OH)₃, which act as effective coagulants under suitable pH conditions [3,8]. The third mechanism involves the interaction of these coagulants with colloidal or dissolved contaminants, facilitating their removal through adsorption, charge neutralization, and floc formation. Finally, the resulting flocs are separated by sedimentation due to their weight, or by flotation, aided by microbubbles of hydrogen gas generated at the cathode that lift the flocs to the surface [4,5,9,10]. Additionally, water electrolysis at the anode produces oxygen (O₂), contributing to the formation of gaseous by-products. It is important to note that the efficiency of these mechanisms can be influenced by operational parameters such as pH, applied voltage, and the composition of the treated effluent [11].

Ozonation is an effective technology for the degradation of recalcitrant organic compounds and color removal in tannery wastewater [12]. Ozone (O₃) acts as a powerful oxidizing agent, reacting directly with double bonds and functional groups [13], and generating hydroxyl radicals (•OH) in aqueous media, which enable advanced oxidation of pollutants as well as the removal of odors and microorganisms [14].

The integration of electrocoagulation and ozonation (EC–O₃) provides a more comprehensive effluent treatment by combining the removal of suspended solids and heavy metals with the degradation of persistent organic compounds and toxicity reduction [15]. This hybrid process exhibits synergistic effects, enhancing the removal of dyes, COD, organic matter, and microorganisms compared to individual methods [16–19]. However, it may generate hard-to-detect byproducts such as aldehydes and carboxylic compounds [7,20], which necessitates further evaluation to ensure environmental safety.

The combined electrocoagulation and ozonation system (EC–O₃) offers significant advantages over conventional technologies by enhancing treatment efficiency and reducing chemical usage. EC generates coagulants in situ, thereby minimizing the production of secondary sludge [3,11], while ozonation oxidizes recalcitrant compounds and improves the biodegradability of the effluent [21]. This synergy enables greater removal of COD, color, and toxicity in shorter treatment times [16,18], with operational costs estimated between 0.4 and 1.5 US$/m³ for EC [22] and approximately 1.25 US$/m³ in tannery effluents [23].

In view of this, an alternative for determining the effectiveness of treatments is the evaluation of effluents through toxicity studies on organisms or bioassays. These studies involve exposing a sample to a representative organism, in which a measurable effect can be observed upon exposure to the test sample [24].

Plant-based bioassays, such as those using Lactuca sativa (romaine lettuce), are effective methods for evaluating parameters such as germination, growth, and biomass. This assay is simple, rapid, and sensitive, allowing for the assessment of the toxicity of both domestic and industrial effluents. Its usefulness has been supported by several studies [25–28].

On the other hand, bioassays with earthworms (Eisenia fetida) are a key tool for assessing soil contaminant toxicity, due to their essential role in soil structure and nutrient availability. This method is notable for its ease of cultivation in various substrates and high sensitivity to contaminants, and is recommended by the OECD for toxicity studies [29,30].

Recent studies highlight the value of bioassays for evaluating industrial effluents. [31] established chloride standards for tanneries using ecotoxicological methods. [28] demonstrated that although electrocoagulation and ozonation reduce contaminants in effluents, toxicity in Lactuca sativa persists at levels comparable to untreated samples. This evidence indicates that traditional physicochemical analyses are insufficient and that bioassays are crucial for assessing actual toxicity.

Despite the effectiveness of electrocoagulation and ozonation in removing contaminants from tannery effluents, their impact on residual toxicity and exposed organisms still requires further investigation due to the possible formation of by-products. This study evaluated the toxicity of effluents treated by integrating both technologies, aiming to determine their suitability for discharge or reuse. Effluent quality was compared with Peruvian regulations for wastewater discharge into sewer systems and for vegetable irrigation, using the bioindicators Lactuca sativa and Eisenia fetida to assess acute and developmental effects. The results seek to contribute to the knowledge of treatments that reduce toxicity and ensure environmental protection.

## Materials and methods

### Tannery process effluents

The effluent samples used in this study were collected during the bovine hide tanning process at the Pilot Tannery Plant of CITEccal Lima, a state institution that collaborated in the project. No formal permits were required for site access or sample collection, as the tanning process was carried out by CITEccal personnel and the wastewater was generated internally for experimental purposes, within the framework of the collaboration established between both institutions. The tanning process involves several sub-stages; therefore, to carry out the treatment, a composite sample was prepared using aliquots taken from the soaking (a process in which surfactants and wetting agents are used to hydrate and clean the hide, as well as to remove the preservation salt), liming and unhairing (a process where lime and sodium sulfide are added to remove the hair attached to the hide), deliming (a cleaning process that also serves to lower the pH by adding ammonium sulfate and sodium bisulfite), bating, pickling, and tanning (processes in which organic acids and chromium-based tanning salts are added).The composite effluent sample was conditioned and stored in containers until treatment.

### Characterization of treated samples

**Fig 1:**
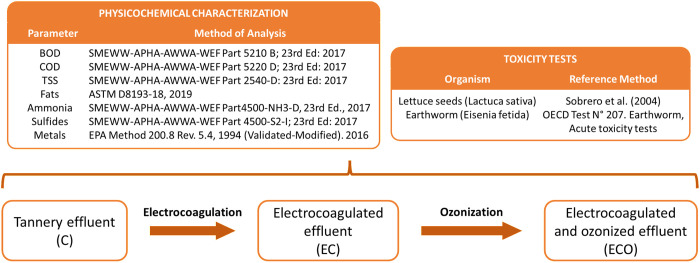
Experimental design and analytical. methods used for the physicochemical and ecotoxicological characterization of tannery effluents.

Raw and treated effluent samples were sent to an accredited laboratory for characterization and determination of the following parameters: Biochemical Oxygen Demand (BOD) (SMEWW-APHA-AWWA-WEF Part 5210 B; 23rd Ed: 2017. Biochemical Oxygen Demand (BOD): 5-Day BOD Test), Chemical Oxygen Demand (COD) (SMEWW-APHA-AWWA-WEF Part 5220 D; 23rd Ed: 2017. Chemical Oxygen Demand, Closed Reflux, Colorimetric Method), Total Suspended Solids (TSS) (SMEWW-APHA-AWWA-WEF Part 2540-D: 23rd Ed: 2017. Solids: Total Suspended Solids Dried at 103–105 °C), ammoniacal nitrogen (SMEWW-APHA-AWWA-WEF Part 4500-NH3-D, 23rd Ed., 2017. Nitrogen (Ammonia). Ammonia – Selective Electrode Method. 2019), turbidity, pH, conductivity, hexavalent chromium content (SW-846 Test Method 7199: Determination of Hexavalent Chromium in Drinking Water, Groundwater, and Industrial Wastewater Effluents by Ion Chromatography), fats, sulfides, and metal content (EPA Method 200.8 Rev. 5.4, 1994 (Validated – Modified). 2016. Determination of Trace Elements in Water and Wastes by Inductively Coupled Plasma-Mass Spectrometry). See Fig 1.

### Treatment by electrocoagulation and ozonation

First, electrocoagulation was employed to remove total suspended solids (TSS) and most of the contaminants, aiming to optimize the efficiency of ozonation. Subsequently, ozonation was applied to reduce sulfides and degrade recalcitrant organic matter in the effluent.

**Fig 2:**
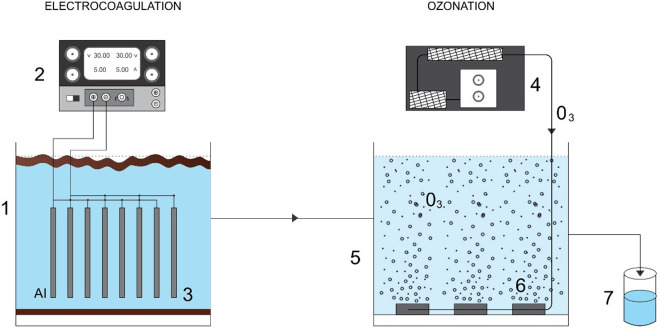
Schematic of the integrated treatment system. (1) Electrocoagulation reactor, (2) Power supply, (3) Aluminum electrodes, (4) Ozonizer, (5) Ozonation tank, (6) Diffusers, (7) Receiving tank.

An electrocoagulation reactor measuring 16 cm (width) × 16 cm (length) × 20 cm (height) and containing 8 aluminum electrodes (10 cm × 10 cm) was used, with the electrodes functioning alternately as anodes and cathodes. The electrocoagulation treatment was carried out at a current intensity of 6 amperes and a treatment time of 24 minutes, which were identified as optimal conditions for the process. Following this, the clarified effluent underwent ozonation, using perforated hose diffusers and an ozonizer capable of generating up to 15 g/h of ozone. The ozonation treatment conditions were set at an ozone feed rate of 12 g/h and a treatment time of 20 minutes (Fig 2). The treated effluent samples were stored for subsequent toxicity assays.

### Evaluation of the quality of treated effluents

The results obtained from the treatment of tannery effluent through electrocoagulation and ozonation processes were compared with the values established in Peruvian regulations for effluent discharge into sewer systems, known as Maximum Allowable Values (VMAs) (D.S. N° 010–2019-VIVIENDA). Additionally, as a complement to the phytotoxicity study, the treatment results were compared with the Environmental Quality Standards (ECAs) for Water (D.S. N° 004–2017-MINAM), specifically under Category 3: Irrigation of vegetables and animal drinking water. These standards serve as indicators of the concentration limits of substances that could contribute to toxicity in plants.

### Battery of bioassays

The test organisms (Lactuca sativa and Eisenia fetida) were exposed to three types of effluent samples: raw effluent (C), consisting of a mixture of effluents from various operations in the leather tanning process; raw effluent treated by electrocoagulation (EC); and raw effluent treated by electrocoagulation followed by ozonation (ECO).

### Evaluation of phytotoxicity with lactuca sativa

Phytotoxicity was evaluated through acute toxicity assays using lettuce seeds (Lactuca sativa). Seeds from the brand BATTLE S.A., variety long romaine light green, with a high germination rate, were used. Analytical-grade reagents were employed, including calcium sulfate dihydrate, zinc sulfate heptahydrate (obtained from EMSURE®), potassium chloride (reagent grade), sodium bicarbonate (reagent grade, ACS, ISO), and anhydrous magnesium sulfate (extra pure, obtained from Scharlau®). Seed germination was carried out in an incubator from the brand VELP, model FTC 90I. The bioassay with Lactuca sativa was conducted following the procedure developed by [26] and described by [28].

The indicators used to evaluate the bioassay results were absolute germination (AG), germination index (GI), and toxicity units (TU).


$$AG=\frac{N_{germ}}{N_{seed}}$$ (1)

$$\;GI=\frac{N_{germ}}{N_{cont}}x\frac{{RL}_{germ}}{{RL}_{cont}}\;$$ (2)

$$\;TU=\frac{1}{{EC}_{50}}\;x\;100$$ (3)

Where N_seed_ es total amount of seeds sown by Petri dish, N_germ_ is the average amount of germinated seeds, N_cont_ is the amount of germinated seeds in the negative control, RL_cont_ is the average root length in the negative control, RL_germ_ is the average amount of root length in each sample and EC_50_ is the median effective concentration, this is the concentration of the sample at which the growth of the root is inhibited at half regarding negative control.

These indicators allow for the evaluation of toxicity levels in a sample. Values of the germination index (GI) equal to zero indicate total inhibition of growth and germination. Values below 0.4 indicate significant inhibition; between 0.4 and 0.8, mild growth inhibition; between 0.8 and 1.2, it is considered that there is no significant effect; and above 1.2, a growth stimulation effect is observed [32]. On the other hand, in the classification of toxicity units, levels are measured by ranges: TU < 0.4 = Class I: No acute toxicity; 0.4 ≤ TU < 1 = Class II: Mild acute toxicity; 1 ≤ TU < 10 = Class III: Acute toxicity; 10 ≤ TU < 100 = Class IV: High acute toxicity; TU ≥ 100 = Class V: Very high acute toxicity [33].

### Bioassay with earthworms

California red worms (Eisenia fetida) were collected from a farm located in Lurín, south of Lima, and transported to CITEccal Lima for conditioning and subsequent use in the bioassay. The conditioning process involved transferring the worms from their original breeding substrate to a base substrate intended for the bioassay, where they remained for a minimum period of 15 days [34]. This base substrate is a sandy loam soil composed of 71% sand, 19% silt, and 10% clay.

The bioassay consisted of exposing the earthworms to a controlled amount of the three types of effluents mentioned, mixed with the substrate. For this purpose, 1-liter cubic plastic containers were used, to which 250 grams of the base substrate employed during the conditioning period and an aliquot of the effluents NC, C, EC, and OZ, respectively, were added. The earthworms were exposed to six effluent aliquots: 20 ml, 40 ml, 60 ml, 80 ml, 100 ml, and 150 ml per container. Additionally, distilled water was added to each container to achieve an approximate moisture content of 70% on a dry basis.

To ensure the quality of the results, a negative control (NC) was included, and the experiment was conducted in quadruplicate. Twenty earthworms were introduced into each container, with prior verification of the presence of a visible clitellum to ensure population homogeneity [34,35].

The survival of the earthworms in each container was evaluated weekly through a thorough inspection, conducted over three consecutive weeks. Additionally, it was verified that the survival rate in the negative control was at least 90% [34,35].

The substrates from the treatments with 100 ml of the NC, C, EC, and ECO samples, after three weeks of exposure, were subjected to FTIR analysis using a SHIMADZU QATR 10 spectrophotometer, via attenuated total reflectance (ATR). The spectra were measured in the range of 4000–400 cm ⁻ ¹.

### Statistical analysis

The acute toxicity assay with L. sativa was conducted using analysis of variance (ANOVA) and Tukey’s test for multiple comparisons to evaluate the presence of significant differences (p-value < 0.05) between samples and concentrations. This analysis was performed using the Real Statistics add-in for MS Excel.

Additionally, a Principal Component Analysis (PCA) and a correlation analysis were developed to explore the relationship between the physicochemical analysis results of the effluent samples and the outcomes of the L. sativa bioassay. Both analyses were performed using R software version 4.4.2 (R Core Team, 2024), utilizing the packages “FactoMineR”, “ggcorrplot”, “ggplot2”, and “ggrepel”.

For the statistical analysis of the E. fetida bioassay, significant differences between the result groups were evaluated, considering the exposure time and sample concentration. The Kruskal-Wallis test was applied, followed by multiple comparisons using the Conover-Iman test with Holm correction. Significant differences were considered at a 95% confidence level (p-value < 0.05). These statistical tests were implemented in R software using the packages “dplyr”, “FSA”, and “PMCMRplus”.

## Results

### Treatment of effluents by electrocoagulation and ozonation

In the first stage, raw tannery wastewater was treated in an electrocoagulation (EC) reactor using aluminum electrodes, applying a current intensity of 5 A for 24 minutes of treatment time. The initial total suspended solids (TSS) concentration decreased from 1820 mg/L to 65 mg/L, corresponding to a removal efficiency of 96.4%. This high efficiency is attributed to optimal floc formation and hydrogen microbubbles generated during the process, which promote flotation and separation of suspended solids [28]. Additionally, removal efficiencies of 30.9% for chemical oxygen demand (COD) and 99% for chromium were achieved. These results are consistent with those reported in the literature for similar systems. [36] obtained 96% TSS removal efficiency when treating tannery effluents, while [37] achieved 85% removal using aluminum electrodes, highlighting the effectiveness of the experimental design employed in the present study.

In the second ozonation stage, preliminary tests were conducted by collecting samples at 10, 20, and 30 minutes. With an ozone dose of 12 g/h, it was observed that the reduction in COD between 20 and 30 minutes was minimal (from 3535 to 3495 mg/L). In the case of sulfides, concentrations close to 0.001 mg/L were reached, indicating a balance point between treatment efficiency and energy consumption. The selection of these parameters was also supported by previous studies on tannery effluents, which identified optimal ozone doses ranging from 7 to 15 g/h and exposure times of up to 30 minutes for the removal of ammonium and COD [4], as well as maximum COD reduction using 10 g/h for 30 minutes [28]. In the final tests, the effluent previously treated by electrocoagulation (EC) was subjected to ozonation (ECO), applying an ozone dose of 12 g/h with a contact time of 20 minutes. This treatment achieved additional removal efficiencies of 10% for chemical oxygen demand (COD) and 99% for sulfides. These results align with previous studies: [38] reported 97.4% sulfide removal efficiency using a higher dose (40 mg/L) and longer treatment time (100 min), while [39] achieved 98% removal by combining ozone with activated carbon.

### Evaluation of the quality of treated effluents

The results obtained from the characterization of samples C, EC, and ECO were compared with the values established in the Maximum Allowable Values (VMAs) and Environmental Quality Standards (ECAs). VMAs were used as the primary criterion, as they represent the permissible limits for effluent discharge by tannery companies. On the other hand, ECAs were used as indicators of potential toxicity for plant development and growth. (Table 1) presents the characterization results for each parameter, highlighting cases where the established standard values were exceeded.

**Table 1:** Characterization results of raw and treated effluents and comparison with environmental standards.

Parameter	Unidad	VMAs	ECAs Irrigation	C	EC	ECO
BOD	mg/L	500.00	15.00	1526.30^a^	1430.00^a^	1048.80^a^
COD	mgO2/L	1000.00	40.00	5608.30^a^	3881.40^a^	3481.40^a^
TSS	mg/L	500.00	–	1820.00^a^	65.00	76.00
Conductivity	mS/cm	–	2.50	22.50^a^	22.00^a^	21.40^a^
Fats	mg/L	100.00	5.00	72.10^a^	33.60^a^	18.90^a^
NH^4+^	mg/L	80.00	–	188.40^a^	294.80^a^	364.10^a^
S^2-^	mg/L	5.00	–	110.81^a^	106.09^a^	0.002
Cl	mg/L	–	500.00	5353.80^a^	5139.18^a^	4628.70^a^
Al	mg/L	10.00	5.00	73.46^a^	19.33^a^	13.89^a^
Ba	mg/L	–	0.70	1.07^a^	0.06	0.04
Cu	mg/L	3.00	0.20	0.20^a^	0.06	0.07
Cr	mg/L	10.00	0.10	107.88^a^	0.45^a^	0.24^a^
Fe	mg/L	–	5.00	5.10^a^	0.98	0.93
Mn	mg/L	4.00	0.20	0.18	0.38^a^	0.09
Pb	mg/L	0.50	0.05	0.05^a^	0.0024	0.0028
Na	mg/L	–	–	5181.48	5058.17	4700.42
Zn	mg/L	10.00	2.00	0.45	0.24	0.32

^a^ Results that surpass ECAs irrigation value or VMAs.

### Phytotoxicity assays with lettuce

The results of the acute toxicity bioassay on Lactuca sativa seeds are presented in (Table 2). In the negative control, a germination rate of 90.0% and a coefficient of variation (CV) of 19.96% were obtained, indicating the validity of the test (germination ≥ 90.0% and CV ≤ 30.0%) [26,40].

**Table 2:** Results of the acute toxicity bioassay on lettuce seeds.

Volume of sample in relation to control hard water volume (%)												
Samples	1		3		10		30		100		EC50	Toxic
	AG	GI	AG	GI	AG	GI	AG	GI	AG	GI	(%)	Units
C	0.98	0.83	0.97	0.51	0.88	0.19	0.00	0.00	0.00	0.00	4.19	23.89
EC	1.00	0.85	0.88	0.67	0.93	0.50	0.90	0.15	0.00	0.00	12.03	8.32
ECO	0.95	0.77	0.95	0.58	0.93	0.45	0.00	0.00	0.00	0.00	8.99	11.12

**Fig 3:**
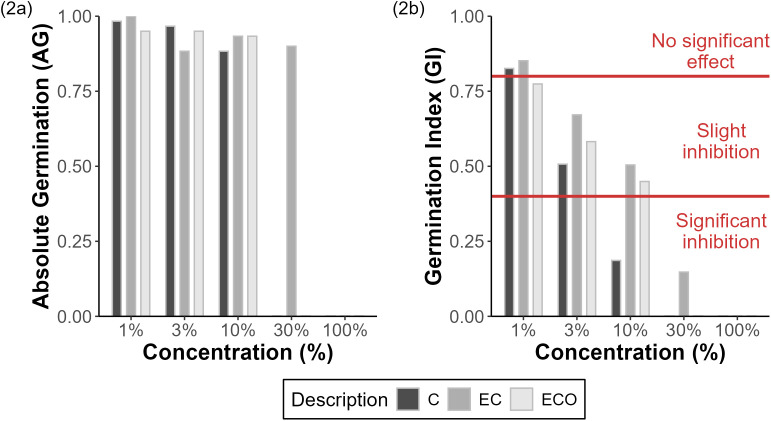
AG and GI for *Lactuca sativa* toxicity test. (2a) Absolute Germination (AG) for each sample at different concentrations. (2b) Germination Index (GI) for each sample at different concentrations with GI classification limits.

According to the germination index (GI) results, presented in (Fig 3), sample C at a concentration of 1% was classified in the “no significant effect” category. However, when the concentration was increased to 3%, a shift to “mild growth inhibition” was observed. At a concentration of 10%, the sample showed “significant inhibition.” Finally, at higher concentrations, total germination inhibition was recorded.

In the case of sample EC, a behavior similar to that of sample C was identified at concentrations of 1% and 3%. However, unlike sample C, at a concentration of 10%, mild growth inhibition was still observed. Additionally, sample EC was the only one that achieved germination and growth at a concentration of 30%, where significant inhibition was recorded. On the other hand, sample ECO showed “mild growth inhibition” at concentrations of 1%, 3%, and 10%. Nevertheless, at higher concentrations, total germination inhibition was evident.

Regarding the analysis by toxicity units (TU), samples C and ECO obtained values above 10, classifying them as Class IV: High acute toxicity. Meanwhile, sample EC was classified as Class III: Acute toxicity. Although samples C and ECO are at the same toxicity level (Class IV), it was observed that sample C exhibited higher toxicity, with an EC50 value of 4.19%, compared to sample ECO, which recorded an EC50 of 8.99%.

Unlike the previous work by [28], the present study identified a higher pollutant load in the initial effluent samples, which explains the higher toxicity values expressed in toxicity units (TU) and the germination index (GI). Regarding the treated samples, the ECO sample, despite showing a higher level of organic matter and sulfide removal compared to the EC sample, exhibited greater toxicity.

Previous studies indicate that the toxicity of complex effluents, such as those generated by the tannery, paper, or petrochemical industries, can be detectable through bioassays but is not always identifiable through physicochemical characterization. Even residual toxicity may persist when physicochemical analyses comply with environmental regulations [41]. This situation highlights the complex nature of tannery effluents, as although treatments such as electrocoagulation and ozonation contribute to reducing the pollutant load, they may also lead to the formation of byproducts, which are often difficult to detect [42–44]. It is possible that a synergy occurs between residual contaminants in the treated effluent and the byproducts formed, making it challenging to identify the specific causes of toxicity [45].

(Table 3) presents a comparison of the results obtained from the characterization of wastewater (C) in this study with those reported in the previous study by [28]. Due to the similar treatment conditions (electrocoagulation and ozonation) in both studies, it is observed that the toxicity values expressed in toxicity units (TU) in the previous study were 5.17, 5.13, and 4.97 for the initial sample, the effluent treated by electrocoagulation, and the effluent treated by electrocoagulation and ozonation, respectively. Likewise, the toxicity values of each sample in the present work are higher than those reported in the previous study. For the present work, it is presumed that the significant difference in phytotoxicity is due to the higher initial pollutant load present in the untreated effluent, primarily organic matter (BOD and COD) and TSS, resulting in higher toxicity.

**Table 3:** Results obtained the present study compared to the previous study.

Values to compare			Results	(Aguilar et al., 2024)
Raw effluent characterization		TSS (mg/L)	1820	980
		COD (mg/L)	5308.3	2961
		BOD (mg/L)	1526.3	561
		Ammonia (mg/L)	188.4	NA
		Turbidity (NTU)	1675	1300
		pH	8.62	7.28
		Conductivity (mS)	22.5	NA
		Total Chromium (mg/L)	107.87	89.52
		Hexavalent Chromium (mg/L)	<0.005	NA
		Fats (mg/L)	72.1	NA
		Sulfides (mg/L)	110.81	NA
Toxicity evaluation		TU (C)	23.89	5.17
		TU (EC)	8.32	5.13
		TU (ECO)	11.12	4.97
Treatment conditions	EC	Ampere (A)	6	7
		time (min)	24	30
	OZ	Dose (gr/h)	10	10
		time (min)	20	30

NA: No available.

In the present work, it is presumed that the significant difference in phytotoxicity is due to the higher initial pollutant load present in the untreated effluent, resulting in higher toxicity. The effluent treated by electrocoagulation showed a significant reduction in toxicity levels, evidencing the decrease in pollutant load identified through physicochemical characterization.

Regarding the effluent treated with ozonation, there is a possibility that, due to the higher residual pollutant load at the time of treatment, byproducts were generated that increased its toxicity. Although this treatment showed positive effects in the removal of substances such as sulfides, total suspended solids (TSS), and chemical oxygen demand (COD), the formation of byproducts during ozonation could explain the observed increase in toxicity [7,42]. Moreover, both treated effluents show a higher pollutant load than the previous study treated effluents.

### Correlation analysis and principal component analysis (PCA)

**Fig 4:**
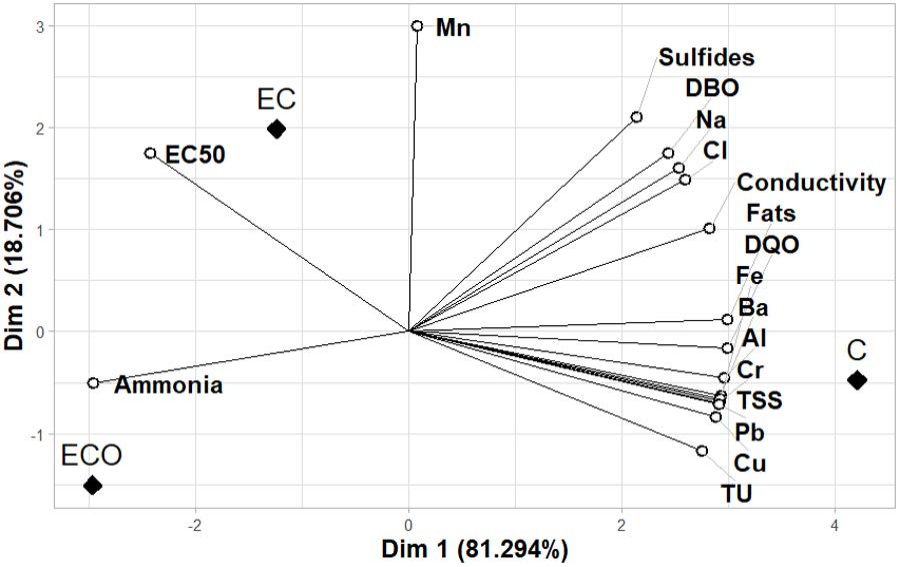
Correlation heatmap for physicochemical parameters characterized. For C, EC and ECO samples.

Both analyses were conducted to clarify the causes of phytotoxicity in the analyzed samples. As a starting point, the results presented in Table 1 were used, where characterization parameters comparable to the VMAs and those exceeding the limits established in the ECAs for vegetable irrigation were identified. This criterion highlights the importance of parameters that must be controlled for effluent discharge by tannery companies and allows for correlating the toxicity assay results with Lactuca sativa, as values exceeding the limits for vegetable irrigation should have a negative effect on seed development. (Fig 4) presents the heatmap of the correlation analysis between the physicochemical parameters characterized in the effluent samples and the toxicity assay results. It is observed that most of the analyzed parameters exceeding the established levels in the standards have an adverse effect, increasing the toxicity of the samples. Among these, the parameters showing less relevance or having a lesser influence on toxicity are biochemical oxygen demand (BOD), sulfides, chlorides, sodium content, and manganese content.

**Fig 5:**
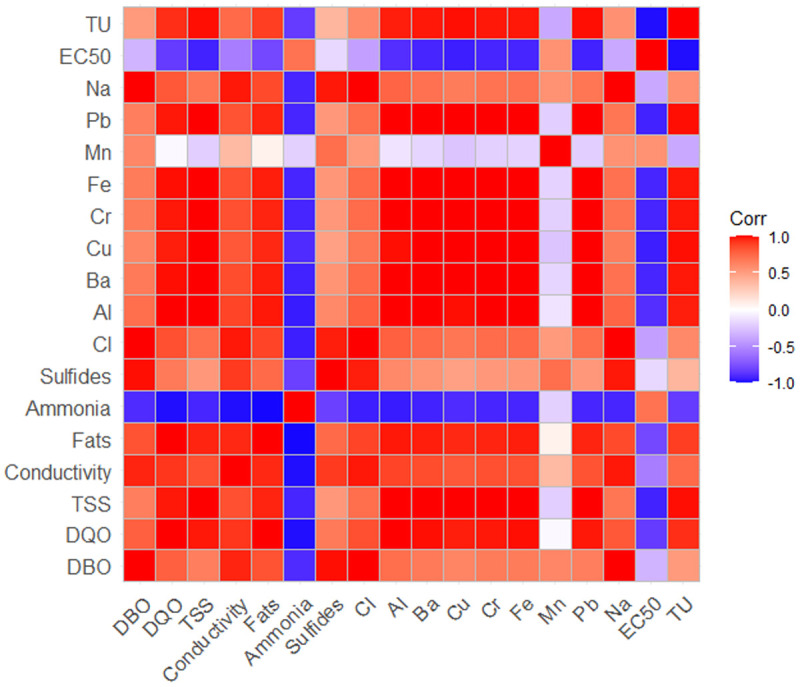
PCA for physicochemical characterization. EC50 and TU of C, EC and ECO samples.

The results of the PCA are presented in (Fig 5) and complement the findings obtained in the correlation analysis [25,46]. The toxicity identified in the samples is primarily related to the content of metals such as chromium, lead, aluminum, and barium, as well as the levels of TSS and COD. These parameters are associated with the results obtained for sample C (raw effluent), which exhibited the highest levels of toxicity and concentration of these contaminants.

In the case of sample EC, which exhibits the lowest toxicity, the analysis associates it with a better result in the EC50. On the other hand, the content of ammoniacal nitrogen is associated with sample ECO. According to the results obtained in both analyses, this parameter appears to be linked to a reduction in the toxicity of the samples. However, this interpretation could be misleading, considering the concentrations found.

The content of ammoniacal nitrogen increased significantly in the treated samples, rising from 188.40 mg NH₄ ⁺ /L in sample C to 294.80 mg NH₄ ⁺ /L in sample EC and 364.10 mg NH₄ ⁺ /L in sample ECO. Despite the observed decrease in toxicity, which suggests a possible relationship between the increase in this parameter and an improvement in toxicity, various previous studies have indicated that ammoniacal nitrogen is a significant source of toxicity identified through bioassays [47,48].

Ammoniacal nitrogen has been identified as a factor responsible for negative effects on plant development, particularly on root growth [49]. Specifically, its impact on Lactuca sativa has been demonstrated, affecting weight, shoot length, and root morphology [50]. Additionally, previous studies have reported adverse toxic effects in bioassays of effluents with even lower concentrations of ammoniacal nitrogen [41,45]. Future research should assess the potential dose-response relationship between ammoniacal nitrogen concentrations and the observed toxicity.

The content of ammoniacal nitrogen also contributes to the toxicity of the samples. Regarding its increase, previous studies applying advanced oxidation processes, including ozonation, for the treatment of tannery effluents have not reported a significant increase or removal of this parameter [51]. However, in works combining electrocoagulation and ozonation, an increase in its concentration has been observed [28,52]. This phenomenon is associated with the degradation of nitrates to nitrites and subsequently to ammoniacal nitrogen.

In particular, [52] identified a correlation between the increase in toxicity in effluents evaluated through *Artemia salina* assays and the increase in ammoniacal nitrogen content in samples treated by electrocoagulation. This issue was mitigated by implementing an integrated treatment system for the removal of ammoniacal nitrogen.

In this sense, the presence of ammoniacal nitrogen in the samples, both initial and treated, also contributes to toxicity. In the correlation analysis, its association with sample ECO is due to the increase in its concentration, which could explain why its toxicity is higher compared to sample EC, where the content of ammoniacal nitrogen is lower.

The formation of byproducts is also related to the mechanism of the ozonation [53,54] which depends on the of the sample, for this study as the pH of the sample was above 8 the ozonation mechanism that may be predominant is through the formation of hydroxyl radicals that destabilize the components in the effluents. The tanning process effluents and tanning process in general involve the processing of skins into leather, the main component of skins is collagen, a macromolecule composed by amino acids that could contribute nitrogen for the generation of ammonia in the effluents, this can be also related to the breakdown of aromatic or aliphatic compounds (present in the tanning chemicals) that contains nitrogen [55].

Other studies have linked toxicity in treated tannery effluents to the presence of residual ammoniacal nitrogen [56]. However, it has also been identified that the primary source of toxicity in these effluents comes from their organic matter content [57]. This supports the results of the present study, as when organic matter is significantly reduced, other substances, such as ammoniacal nitrogen, can express their toxicity more intensely. However, it is not discarded that the increase in the toxicity could be related to the degradation and generation of other byproducts (such as aldehydes or carboxylic constituents) that were not analyzed in the samples and were beyond the scope of this work, so it is recommend for further studies to consider them for analysis [20,42].

### Bioassay with earthworms

**Fig 6:**
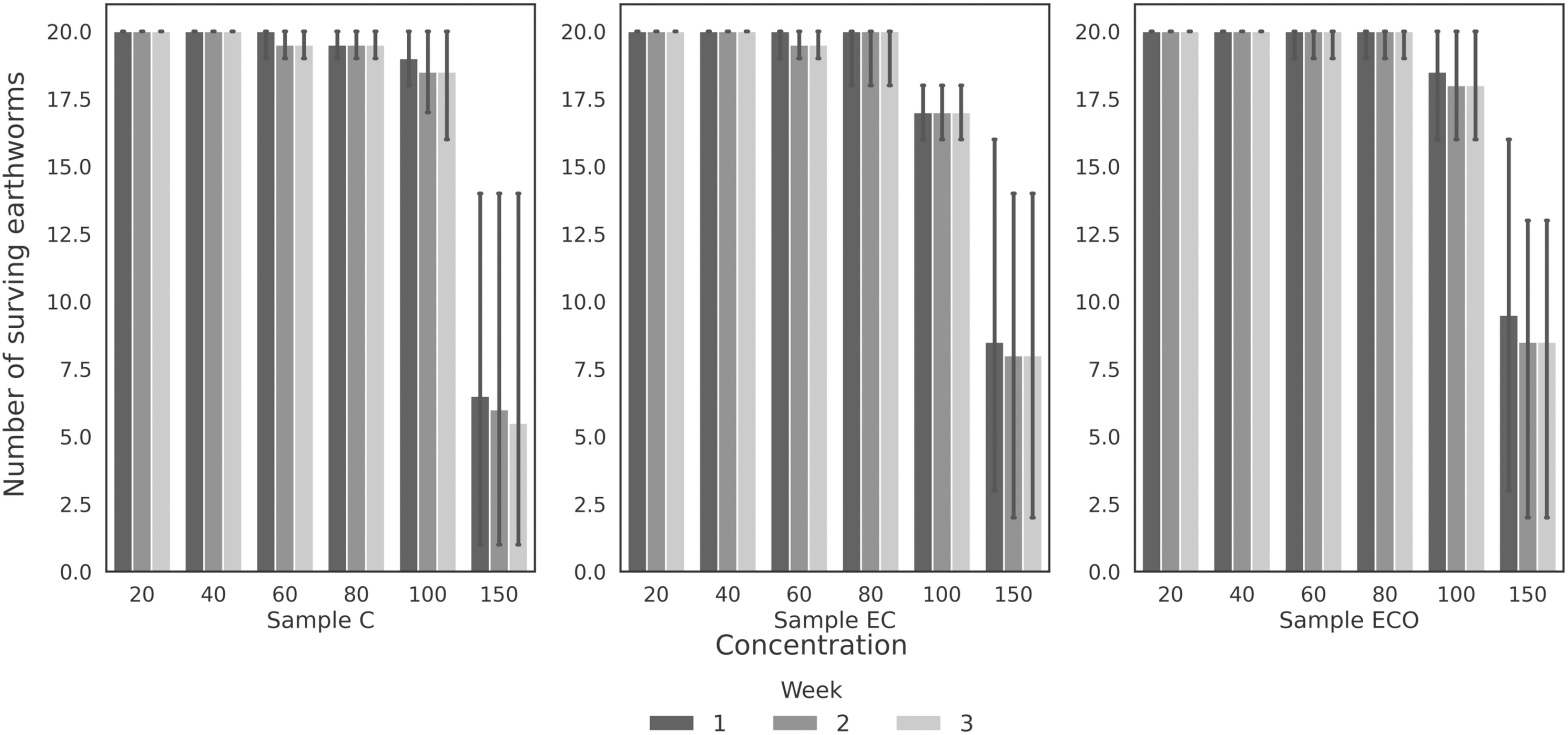
Number of surviving earthworms per concentration. From left to right, the results are shown for samples C (raw effluent), EC (raw effluent treated with electrocoagulation), and ECO (raw effluent treated with electrocoagulation and ozonation).

In the bioassay with earthworms, the negative control (NC) reported a survival rate above 90%, meeting the control criteria established in the OECD and EPA guidelines. (Fig 6) presents the results of the bioassay with E. fetida exposed to different concentrations of the effluent samples.

Regarding the analysis of samples C, EC, and ECO, no significant differences in earthworm survival were observed up to concentrations of 80 mL. However, in treatments where 100 mL and 150 mL of the sample were added, a significant decrease in the earthworm population was recorded from the first week compared to the other concentrations (p-value < 0.05 according to the Kruskal-Wallis test and p-value < 0.05 for the Conover-Iman test).

Similarly, a comparison was made between the weeks of exposure for the 100 mL and 150 mL concentrations of each sample. In this analysis, no significant decrease in the count of surviving earthworms was identified (p > 0.05, according to the Kruskal-Wallis test). This suggests that, after the mortality observed during the first week, the earthworms adapted and showed some resistance to the compounds present in the effluents during the remaining evaluation period.

Earthworms have the ability to stabilize complex organic compounds and modify physicochemical parameters, which could explain the stability in their survival throughout the exposure period [58]. However, it would be advisable to extend the study duration and incorporate additional toxicity indicators to evaluate other potential effects on the test organisms.

Tannery effluents typically contain nitrates, sulfates, sulfides, chlorides, chromium, ammoniacal nitrogen, surfactants, and organic dyes, among other compounds [30,59]. The composition and concentration of these contaminants vary depending on the type of skin processed. Chromium, total dissolved solids, and sodium have shown a strong correlation with toxicity in test organisms [59].

Due to their adverse effects on various aquatic species, these effluents have been classified as very toxic or highly toxic, even at low concentrations (1.0% − 4.5%), affecting organisms such as Daphnia magna, Physa venustula, and Xenopus laevis. However, the results obtained in this study with earthworms present a contrast, possibly because their exposure to these compounds is indirect and mediated by the absorption of the effluent into the soil. In this context, the exposure is more controlled and gradual compared to that of aquatic organisms, which are in direct contact with the effluent. Future research should consider conducting additional toxicity tests using aquatic organisms representative of freshwater ecosystems, such as microalgae, crustaceans, or fish embryos, to obtain a more comprehensive evaluation of the treated effluents’ environmental impact. These tests would complement the findings obtained with Lactuca sativa and Eisenia fetida and help better predict potential ecological risks in receiving water bodies.

**Fig 7:**
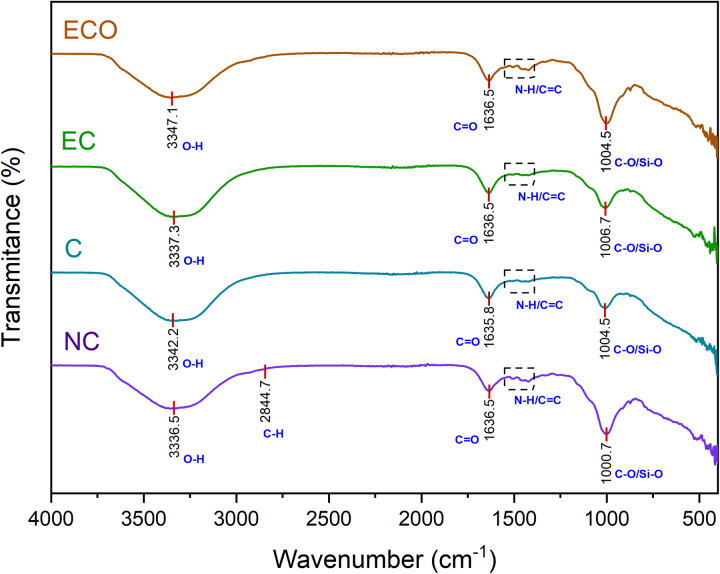
FTIR spectra for the NC. Corresponding to garden soil, and for the treatments with 100 mL aliquots of samples C, EC, and ECO mixed with the NC, after exposure to earthworms for three weeks.

On the other hand, although the toxicity of effluents is expected to decrease alongside the reduction in physicochemical parameters after treatment, these parameters alone do not provide information about the effects on organisms. Likewise, they do not allow for the evaluation of potential synergistic or antagonistic interactions between residual compounds after treatment [30].

(Fig 7) shows the results of the FTIR analysis performed on the soil samples obtained from the bioassay. In the NC sample, absorption bands were identified in the range of 3500–3200 cm ⁻ ¹, associated with O–H functional groups, which can be attributed to the presence of adsorbed water or clay minerals. Additionally, bands between 2855 and 2804 cm ⁻ ¹ were observed, related to C–H bonds (alkanes) characteristic of organic matter. In the range of 1697–1600 cm ⁻ ¹, bands corresponding to C = O groups (carbonyls) of organic compounds present in the soil, such as humic acids, were detected. Furthermore, bands between 1544 and 1400 cm ⁻ ¹ were identified, associated with N-H/C = C groups (amines and aromatic compounds). Bands around 1000 cm ⁻ ¹ were also observed, related to C-O/Si-O bonds, typical of silicates, quartz, and clays [60–62].

In the C sample, a change in the intensity of the bands in the region of 1000−900 cm ⁻ ¹ was observed compared to the NC sample. These bands can be attributed to C-O groups, present in compounds such as alcohols, ethers, esters, and carboxylic acids [63]. The decrease in intensity suggests that these compounds may have been degraded or transformed during the process.

In the EC sample, a decrease in the intensity of some bands present in the C sample was observed, indicating that the electrocoagulation process may have removed or degraded certain organic or inorganic compounds present in the effluent. On the other hand, in the ECO sample, some of the changes observed in EC were maintained, but an intensification of certain bands was detected, particularly in the region of 1000 cm ⁻ ¹. This increase in intensity could be associated with the oxidation of organic matter or the formation of new functional groups that absorb in this region of the infrared spectrum.

Despite these observations, the toxicity of the samples did not show significant differences in the earthworms, which could be attributed to their natural resistance or their ability to degrade contaminants. However, a more detailed study including biomolecular indicators in the earthworms could provide more precise information on the impact of the effluents. This approach would allow for a deeper evaluation of whether vermifiltration could constitute an alternative and/or complementary technology to conventional physicochemical treatments.

## Conclusions

This study demonstrates the effectiveness of an integrated electrocoagulation (EC)-ozonation system for treating tannery wastewater. The EC process achieved removal efficiencies of 96.4% for total suspended solids (TSS), 30.9% for chemical oxygen demand (COD), and 99% for total chromium. Subsequent ozonation of the treated effluent provided additional removal of 10% COD and eliminated 99% of present sulfides.

Regarding toxicity evaluated through bioassays with Lactuca sativa and Eisenia fetida, a significant reduction in toxicity was observed between the initial effluent and the samples treated by electrocoagulation and ozonation in the case of L. sativa. However, the ozonated sample exhibited higher toxicity compared to the electrocoagulation-treated sample. This result was associated with the generation of ammoniacal nitrogen during treatment, whose concentration increased significantly in each treated sample. Additionally, the possible formation of byproducts during ozonation, which could contribute to the increase in effluent toxicity, cannot be ruled out.

In the case of the E. fetida assay, low acute toxicity was determined for all samples. These results suggest the feasibility of treating these effluents using techniques such as vermicomposting and vermifiltration. However, further study is necessary through the identification of other potential effects on the test organisms using biomarkers, which would allow for determining the optimal treatment volume based on the observed effects.

This research not only highlights the potential and limitations of electrocoagulation and ozonation in the treatment of tannery wastewater to meet environmental standards, whether individually or integrated, but also emphasizes the importance of considering toxic effects on organisms as a key criterion in environmental quality assessment, especially for this type of effluent, characterized by its high complexity. Furthermore, it highlights the persistence of ammoniacal nitrogen and the need to investigate complementary treatment strategies.

To strengthen the understanding of the ecotoxicological implications of this integrated treatment, future studies should aim to elucidate the dose-response relationship between ammoniacal nitrogen concentrations and observed toxicity. It is also essential to characterize the nature and potential impact of others by-products formed during ozonation. Additionally, expanding the ecotoxicological evaluation to include other aquatic bioindicators, may help capture a broader range of potential effects and support the definition of complementary treatment strategies for tannery effluents.

## Supporting information

S1 Dataset Bioassays results dataset. Results are presented in Microsoft Excel, the first sheet “L.sativa” displays results for Lactuca sativa bioassay, expressed on measurements of radicle elongation in centimeters. The results of Eisenia fetida bioassay are displayed on sheets 2, 3, and 4, which are called “E.fetidaS1”, “E.fetidaS2”, and “E.fetidaS3” respectively, these are expressed on the count of surviving earthworms per test, where each sheet is the result for each week that the study lasted.
(XLSX)
